# Photocatalytic Degradation of Xylene by Carbon Quantum Dots/Clinoptilolite Composites

**DOI:** 10.3390/ma16155243

**Published:** 2023-07-26

**Authors:** Shuguang Zhu, Chun Cheng, Li Meng, Pengyu Zhang, Bai Sun

**Affiliations:** 1 Engineering Research Center of Building Energy Efficiency Control and Evaluation, Ministry of Education, Anhui Jianzhu University, Hefei 230601, China; zhushuguang@ahjzu.edu.cn; 2 Energy Saving Research Institute, Anhui Jianzhu University, Hefei 230601, China; 3 Key Laboratory of Water Pollution Control and Wastewater Recycling of Anhui Province, Hefei 230601, China; hayamimio@outlook.com (C.C.); ml572436870ml@outlook.com (L.M.); zpy110817@163.com (P.Z.); 4 School of Environmental and Energy Engineering, Anhui Jianzhu University, Hefei 230601, China

**Keywords:** CQDs, clinoptilolite, xylene, CQDs/clinoptilolite photocatalyst, hydrothermal method

## Abstract

In this work, a series of clinoptilolite composites decorated with carbon quantum dots (CQDs/clinoptilolite) with hierarchical pore structures was demonstrated that exhibits good photocatalytic performance for the removal of xylene. The technique for the attachment of carbon quantum dots to clinoptilolite was prepared by a hydrothermal method in this study. The structural features were confirmed by SEM, TEM, EDS, XRD, BET, XPS, and solid diffuse reflection measurements, while the degradation mechanism was investigated by adding a trapping agent into the nanocomposites. The introduction of CQDs promoted the separation of photogenerated electrons and holes as well as the generation of reactive radicals, which effectively improved the light utilization and even increased the degradation rate of xylene by 73% at the optimal state. The photocatalytic test was conducted under a different dwell time, catalyst dosage, initial concentration, and illumination intensity. The results showed that the degradation rate of xylene by the CQDs/clinoptilolite catalyst reached 97.4% under the optimal reaction conditions (the catalyst was Catalyst No. 2, the residence time was 90 s, the initial concentration was 2.5 g/m^3^, the light intensity was three lamps for irradiation, and the catalyst dosage was 0.05 g). In addition, the degradation efficiency of the CQDs/clinoptilolite photocatalyst still reached 78% after eight consecutive catalytic regeneration cycles. This work sheds new light on the degradation of xylene.

## 1. Introduction

People generally spend more than 80% of their time in indoor environments such as homes, offices, workshops, and even cars. Therefore, human health advocates as well as environmentalists have focused a substantial portion of their attention on indoor air quality and the control of indoor air pollution because of their effects on human health and on the environment. Volatile organic compounds (VOCs) are well known as indoor air pollutants that have toxic effects on the human body [1,2,3]. They are usually emitted from construction materials, office equipment, and decoration paint. Aromatic hydrocarbon is one of the main volatile organic compounds (VOCs) in indoor air, among which xylene is the representative aromatic hydrocarbon. The removal of xylene is of great significance for indoor air purification [4,5,6].

Various technologies can be used for the removal of xylene such as adsorption, catalytic oxidation, membrane filtration, or electrochemical methods [7,8,9]. From the technological future trends of the literature studies, it can be concluded that one of the most desirable and environmentally friendly technologies is the photocatalytic oxidation for xylene removal [10,11]. As a typical porous material, zeolites can be divided into natural zeolites and synthetic zeolites. Natural zeolite is cheap and abundant in content, so the use of natural zeolite, especially clinoptilolite, in synthesis photocatalysts has increased [12,13]. Photocatalysts were prepared by titanium dioxide doped on clinoptilolite nanoparticles for the simultaneous degradation of 2,4-dichlorophenoxyacetic acid (2,4-D), and a 2-methyl-4-chlorophenoxyacetic acid (MCPA) mixture under ultraviolet and sunlight irradiations [14]. A novel ternary BiOCl/TiO_2_/clinoptilolite nanocomposite was prepared by a facile hydrothermal route combined with a water bath precipitation, which were used to degrade sodium isopropyl xanthate (SIPX) under visible light, and the results showed that the degradation rate could exceed 90% [15]. Carbon quantum dots (CQDs) are zero-dimensional (0D)-like carbon-based materials with low resistance, good chemical stability, and strong light trapping ability [16,17]. As an electron acceptor, CQDs can effectively inhibit the recombination of electron-hole pairs when combined with other photocatalysts [18]. For example, much better photocatalytic activity in the degradation of Reactive Black_5_ (RB_5_) was achieved by TiO_2_/graphene quantum dots (GQDs) under sunlight irradiation compared with pure TiO2 nanoparticles (NPs) due to its lower band gap (2.13 eV) and electron/hole recombination rate [19]. The CQDs possess exceptional optical and electrical properties compared with their counterparts, such as graphene oxide and carbon nanotubes (CNTs) [20]. The CQDs are synthesized by simple synthesis routes and possess outstanding physicochemical and excellent optoelectronic properties, which can be used for the photocatalysis [21]. 

Based on the above discussion, a series of CQDs/clinoptilolite composite materials were synthesized to achieve the photocatalytic degradation of xylene driven by ultraviolet light. The composite of clinoptilolite loaded with carbon quantum dots was prepared by a hydrothermal method, and the characterization of the material was analyzed to determine the production of the composite [22,23,24]. The effects of dwell time, catalyst dosage, initial concentration, and light intensity on the degradation of xylene gas were investigated. The photocatalytic degradation mechanism of carbon quantum dots in CQDs/clinoptilolite composites was investigated by radical capture experiments and characterization analysis of the composites [25,26,27]. The aim of this study is to achieve the harmless removal of xylene in a highly efficient and low-cost way.

## 2. Materials and Methods

### 2.1. Chemicals

Natural clinoptilolites were acquired from Liaoning province, China. Glucose (C_6_H_12_O_6_, AR), sodium hydroxide (NaOH, AR), hydrochloric acid (HCl, AR), ethanol (C_2_H_5_OH, AR), and xylene (C_8_H_10_, AR) were purchased from Sinopharm Chemical Reagent Co., Ltd. (Shanghai, China).

Deionized (DI, H_2_O) water was provided by the FST-TOP-A24 Model number manufactured by Fusite Instrument Equipment Co., Ltd. (Shanghai, China). The chemicals used in the experiment were analytically pure and could be used without further purification.

### 2.2. Preparation of Catalysts

Clinoptilolite composites catalyst decorated by the CQDs was prepared in solution by hydrothermal method. Due to the coarse particles of the original clinoptilolite, the powders needed to be pretreated before preparation. Firstly, the clinoptilolite powders were screened with 300 mesh sieves after grinding and crushing. The screened clinoptilolite powder was washed several times with distilled water to clarify the supernatant. Ultrasonic cleaning was carried out for 1 h, and then the cleaning was repeated several times. Then put the cleaned clinoptilolite in the oven and dried it at 70 °C for 6 h. Finally, the dried clinoptilolite material was stored in a sealed bag. Weighed three parts of 5.00 × 10^−3^ mol clinoptilolite and put them into three 50 mL reaction kettle liners, Then accurately pipetted 25 mL of deionized water into the above reaction kettle, stirred the solution with a glass rod, added 0.00 mol, 6.88 × 10^−3^ mol, and 1.38 × 10^−2^ mol glucose into the three mixed solutions, stirred again to dissolve them, and finally added 5 mL of 4 M NaOH solution to adjust the PH to 14, tightened the reaction kettle, and put it into an oven. The reaction conditions were set as follows: temperature 165 °C, reaction time 24 h. After the reaction was finished, dropped to room temperature, took out the reactor, filtered the black and brown solution in the reactor (with 22 μm organic filter membrane), and cleaned the lining of the reactor in time, rinsed with deionized water and 95% ethanol until the filtrate was colorless. The samples were wrapped in filter paper and then dried at 70 °C for 4 h. The resulting samples were named the original zeolite, catalyst No. 1, and catalyst No. 2. The prepared components and dosages of the three materials are shown in Table 1.

### 2.3. Catalyst Characterization

Scanning electron microscopy (SEM, S4800, Hitachi, Tokyo, Japan) was used to observe the surface morphology of the catalyst, and the elemental composition of the catalyst was determined by Energy Dispersive Spectrometer (EDS). The size of CQDs was observed by a transmission electron microscope (TEM, JEM-F200, Japan Electronics Co., Ltd., Tokyo, Japan). The crystal structure of the catalysts was tested by an X-ray diffraction analysis (XRD, PANalytical, Almelo, The Netherlands) with Cu Kα radiation. The X-ray photoelectron spectroscopy (XPS, Thermo Fischer ESCALAB Xi^+^, Waltham, MA, USA) was used to determine the chemical bonding environment. Moreover, Brunauer–Emmett–Teller (BET, TriStar II, Micromeritics Instrument Corporation, Norcross, GA, USA) was applied at 77 K to determine the specific surface areas based on N_2_ adsorption–desorption isotherms.

### 2.4. Evaluation of Photocatalytic Activity

**Figure 1:**
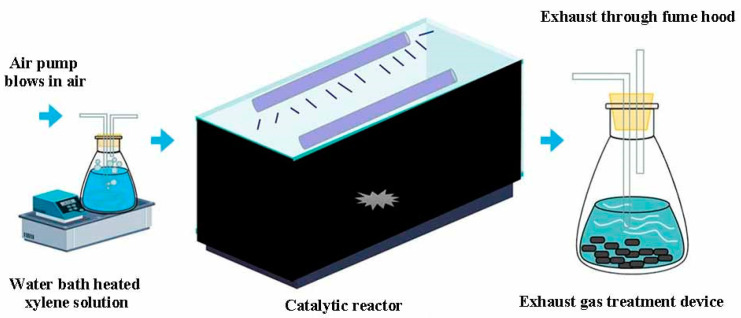
Experimental device diagram.

The reactor used in the experiment consists of a xylene gas distribution system, a control gas circuit, a mixing buffer zone, a photolysis area, a tail gas treatment device, and other parts. Xylene is introduced into the reaction system by means of aeration, and relative humidity and initial xylene concentration are adjusted by controlling the gas circuit. After the xylene, air, and water vapor are fully mixed in the mixing buffer zone, they enter the photolysis reaction zone and then enter the exhaust gas treatment unit. For the catalytic experiments, a homemade reactor model was used, and the sample was placed into the center of the reactor. Before the catalytic experiments, N_2_ was passed through the sample at a rate of 100 mL/min for 1 h at 100 °C to remove the adsorbed water. Direct air pump was used to mix air with xylene vapor produced by xylene generator to simulate factory waste gas, the initial mass concentration and initial flow rate of xylene were controlled by a mass flow meter, and the outlet gas was connected to a VOC gas detector for detecting the xylene concentration at the outlet. As shown in Figure 1, the effective volume of the UV photolysis area was 0.5 L. Capillary tubes were used to make the gas distribution uniform, and the number of vacuum UV lamps filled in the middle was selected according to the experimental variables.

VOC-608 portable gas detector was used to detect the gas concentration in the sample port before and after the reactor. The sensitivity of VOC-608 portable gas leak detector is better than 1 ppm and can be used for VOC gas, combustible gas detection, etc., and can achieve long-term monitoring.

## 3. Results and Discussion

### 3.1. Structural Characteristics of the Catalysts

**Figure 2:**
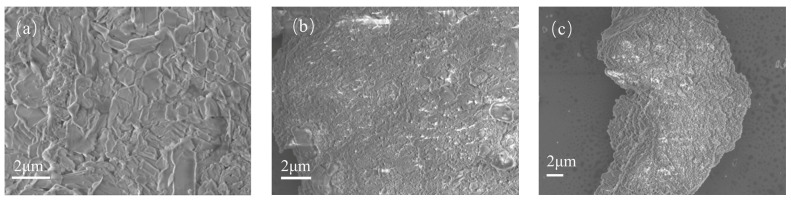
SEM images of (**a**) the original zeolite, (**b**) Catalyst No. 1, (**c**) Catalyst No. 2.

**Figure 3:**
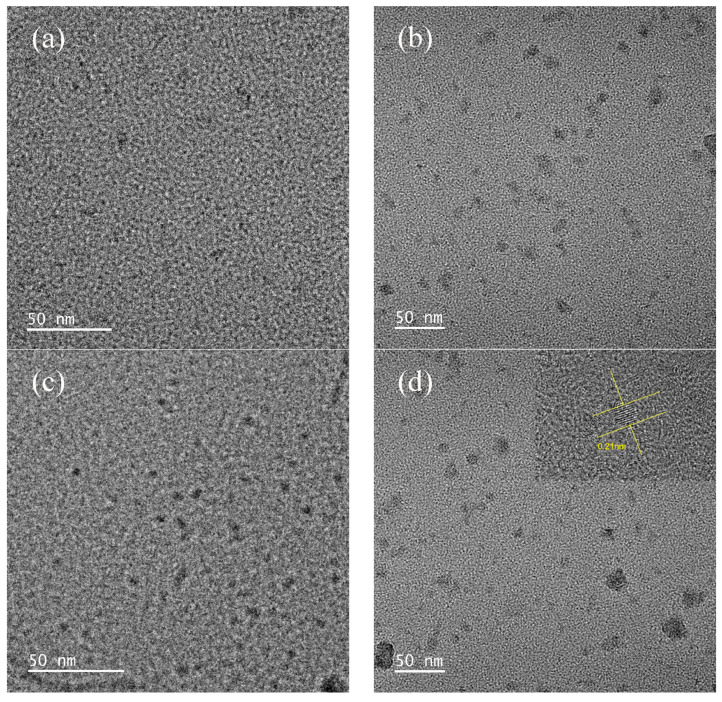
TEM images of (**a**) Catalyst No. 1, (**b**) Catalyst No. 1, (**c**) Catalyst No. 2, (**d**) Catalyst No. 2.

SEM and TEM were utilized to characterize the morphology and microstructure of the catalysts. From Figure 2a, the original zeolite is massive, with a flat and smooth surface and a regular and tight structure. From Figure 2b,c, there are lamellar structures relatively uniformly covering the surface of the original zeolite, which are tentatively estimated to be carbon layers. The carbon layer obtained under the condition of 1.38 × 10^−2^ mol of glucose is more uniform in structure relative to the samples prepared under the condition of 6.88 × 10^−3^ mol of original zeolite, which may be due to the relatively higher amount of glucose and more complete encapsulation. The higher the content of the carbon layer compounded on the surface of the original zeolite, the denser the carbon layer is [28]. To confirm the successful loading of CQDs on clinoptilolite, the prepared materials were characterized by TEM. Figure 3a,b show the results of the TEM imaging experiments for Catalyst No. 1; Figure 3c,d show the results of the TEM imaging experiments for Catalyst No. 2. From the figure, it can be observed that the surfaces of both materials are covered with uniform carbon dots. The number of carbon dots increases significantly with the increase in glucose dosage. Combined with the high-resolution lattice phase of the samples, it can be concluded that the lattice spacing of carbon dot particles is 0.21 nm, which is consistent with the lattice spacing size of carbon quantum dots reported in the literature [29]. The TEM imaging results indicate that clinoptilolite was successfully loaded with CQDs during the preparation of the composites using a one-step hydrothermal method.

**Figure 4:**
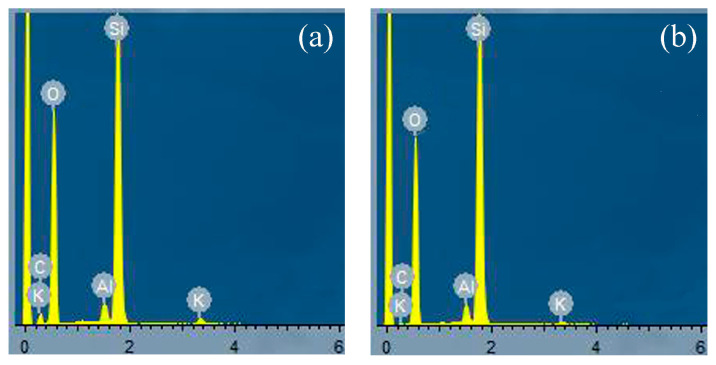
EDS images of (**a**) Catalyst No. 1, (**b**) Catalyst No. 2.

From the EDS energy spectra in Figure 4a,b, the elements of the material are mainly C, K, O, Si, and Al, and no other elements are doped. According to the literature, the carbon content of clinoptilolite is very low, and no other substances are observed on the surface of clinoptilolite, so it is presumed that the CQDs may be loaded on the surface of clinoptilolite. Table 2 shows the elemental content of Catalyst No. 2.

**Figure 5:**
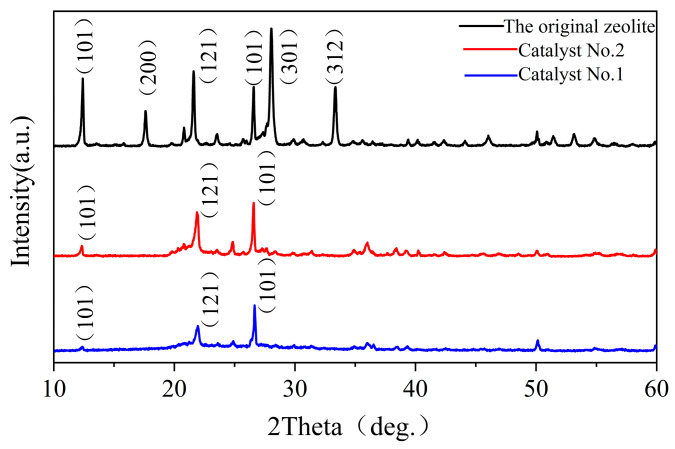
XRD pattern of catalyst No. 1 and catalyst No. 2 and the original zeolite.

To further corroborate the successful preparation of CQDs on the surface of clinoptilolite, the composites were characterized by XRD. Figure 5 shows the XRD patterns of the different catalysts. The diffraction peaks of the composites at 2θ = 22.1°, 26.9°, 28.1°, and 30.1° correspond to the three standard cards (JCPDS No. 39-1383) [30], (JCPDS No. 86-1629) and (JCPDS No. 89-3434), which are compatible with the characteristic peaks of clinoptilolite. The characteristic peaks of SiO_2_, C, Al_2_Si_2_O_5_(OH)_4_, and other substances also matched with those of clinoptilolite. The results showed that the structure of clinoptilolite remained basically unchanged after treatment, indicating that the crystal skeleton of clinoptilolite is intact with good material crystallinity. A small bulge around 2θ = 23° was found in the profiles of Catalyst No. 1 and Catalyst No. 2, which was not present in the original zeolite. The combination of the XRD characterization of amorphous carbon and the synthesis of carbon quantum dots on the surface of clinoptilolite in the Catalyst No. 1 and Catalyst No. 2 composites did not cause any structural damage to the clinoptilolite. It was also found that several characteristic peaks disappeared in the hydrothermally treated Catalyst No. 1 and Catalyst No. 2 compared to the original zeolite XRD patterns. As the amount of glucose increased, the characteristic peaks of carbon became more intense and the intensity of the characteristic peaks of clinoptilolite decreased.

**Figure 6:**
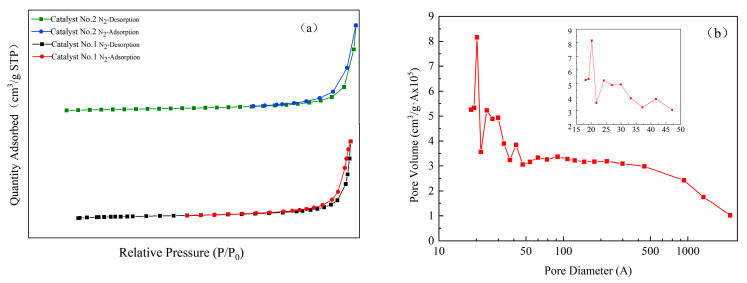
(**a**) N_2_ adsorption and desorption isotherms, (**b**) pore diameter distribution of Catalyst No. 1.

In order to test the adsorption performance of the prepared composites, the specific surface area and pore size of the material were further analyzed. The N_2_ adsorption and desorption tests were carried out at 77 K. The specific surface areas of Catalyst No. 1 and Catalyst No. 2 were 6.1522 m^2^/g and 5.7169 m^2^/g, respectively. Figure 6a shows that the N_2_ adsorption and desorption isotherms for Catalyst No. 1 and Catalyst No. 2 are V-shaped curves, which are characteristic of the curves that should be exhibited by mesoporous materials [31,32]. Figure 6b shows the pore size distribution of Catalyst No. 1, and the pore size of the material is mainly distributed between 15~100 nm. It indicates the presence of pores in the composites, which are mainly mesoporous and macroporous in size. Because of the small specific surface area of the prepared composites and the presence of mainly mesopores and macropores, the adsorption performance of the composites for liquids and gases was not satisfactory.

**Figure 7:**
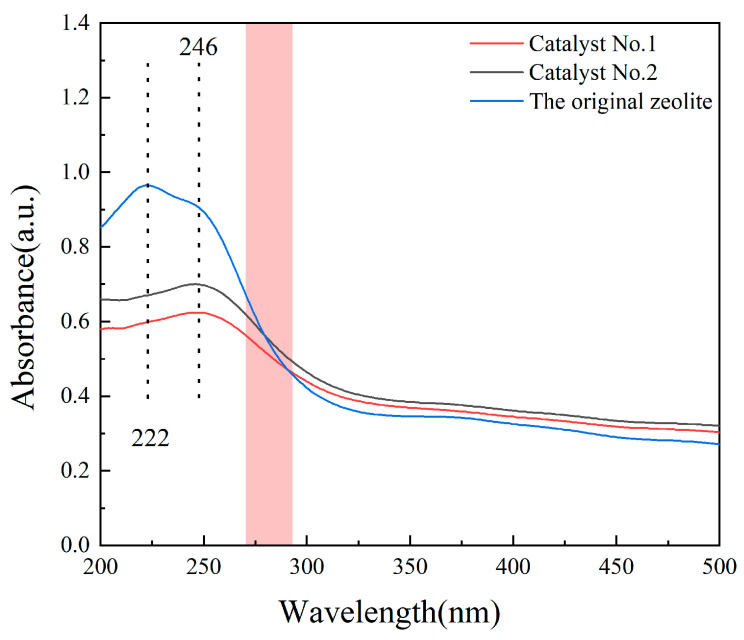
Solid diffuse reflectance spectrogram.

Solid diffuse reflectance analysis was also performed on the composites to confirm the synthesis of carbon quantum dots. Figure 7 shows the solid diffuse reflectance patterns of Catalyst No. 1, Catalyst No. 2, and the original zeolite. The absorption peak of the original zeolite is at 222 nm, and the peak at 222 nm disappears after the reaction with glucose. Silicon dioxide has no absorption peak, but there is an absorption peak at 246 nm in the figure. According to the literature, a distinctive feature of carbon quantum dots is the strong absorption peak in the UV region around 250 nm, and then combined with TEM and XRD analysis, it was determined that the characteristic peak here is the absorption peak of carbon quantum dots [33,34]. Combining the above characterization analysis, it can be concluded that CQDs/clinoptilolite composites were successfully prepared and CQDs were uniformly dispersed on the surface of clinoptilolite.

**Figure 8:**
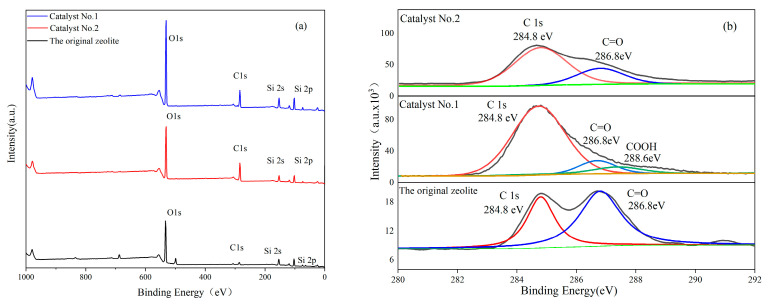
XPS spectra of (**a**) survey scan, (**b**) C 1 s of the original zeolite, Catalyst No. 1 and Catalyst No. 2.

Figure 8a,b show the XPS spectra of the original zeolite, Catalyst No. 1, and Catalyst No. 2. The signals of C, O, Si, and Al can be clearly observed in the XPS full spectrum of Figure 8a, indicating that the CQDs/clinoptilolite composites are composed of C, Al, and SiO_2_. Since the spectra of the two treated composites and the original zeolite are very similar, except that the intensities of C and O are higher than those of the original zeolite, and based on the percentage change in the elemental atoms in Table 3 as well as the characterization and analysis of the materials in the previous section, the presence of a loading of CQDs can be inferred. The high-resolution XPS spectra of carbon elements were fitted and analyzed, and the characteristic peaks at binding energies of 284.8 eV and 286.8 eV for Catalyst No. 2 in Figure 8b were attributed to C 1 s and C=O, respectively. The characteristic peaks at binding energies of 284.8 eV, 286.8 eV, and 288.6 eV in Catalyst No. 1 were attributed to C 1s, C=O, and carboxyl groups, respectively [35]. The characteristic peaks appearing at binding energies of 284.8 eV and 286.8 eV for the original zeolite were attributed to C 1s and C=O, respectively, while the intensity was much lower compared to Catalyst No. 1 and Catalyst No. 2.

### 3.2. Influence of Reaction Conditions on the Degradation Effect

Various disturbing factors as well as the nature of the catalyst itself can affect the catalytic process. This experiment analyzed the possible influencing factors, including residence time, catalyst dosage, initial concentration, and light intensity.

**Figure 9:**
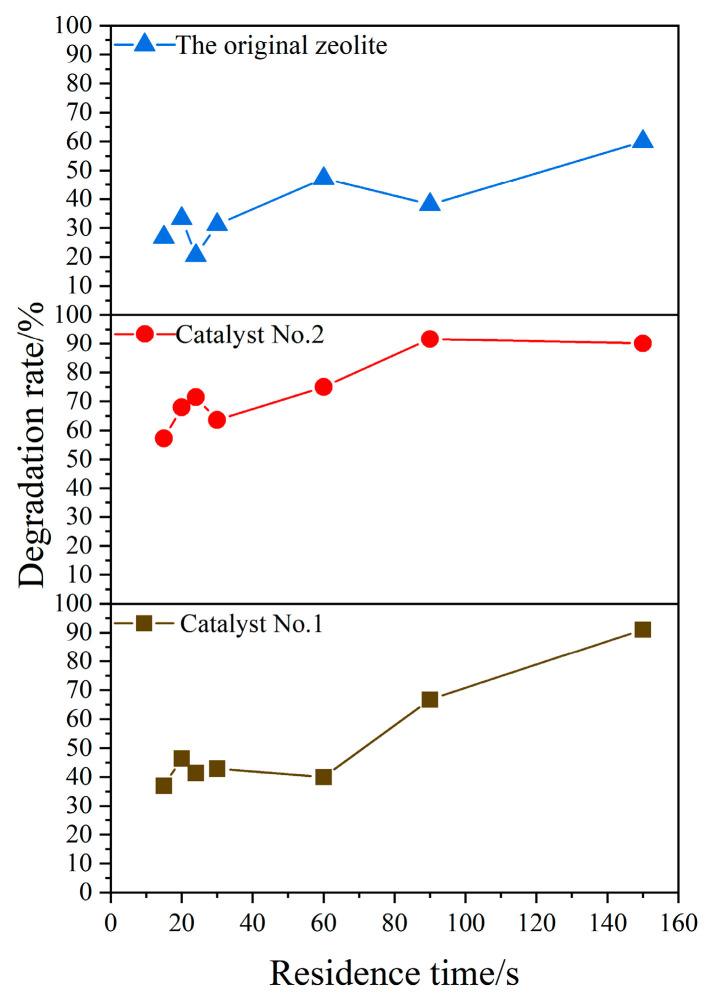
Influence of residence time (T = 20 °C, UV = 2 lamps (a lamp power of 8 w), catalyst dosage = 0.05 g).

The residence time reflects the contact reaction time between the catalyst and the gas-phase pollutant, and is one of the important process parameters affecting the photocatalytic degradation rate [36]. The CQDs/clinoptilolite composites were studied under other optimal conditions for the single factor of residence time, as shown in Figure 9. The photocatalytic degradation of xylene was carried out at different residence times, measure inlets, and outlet xylene gas concentrations separately. When Catalyst No. 1 was used, the degradation rate showed an increasing trend with the extension of time, and the degradation rate reached as high as 90% when the residence time was 90 s. When Catalyst No. 2 was used, the degradation rate reached a maximum of 92% at a residence time of 90 s. In contrast, the degradation rate tended to be flat when the original zeolite was used as the catalyst, and all of them were lower than 56%. The results showed that the degradation rate of xylene was directly related to the residence time, and the longer the residence time, the higher the degradation efficiency. Catalyst No. 2 had the best degradation efficiency, which was significantly improved compared with the original zeolite.

**Figure 10:**
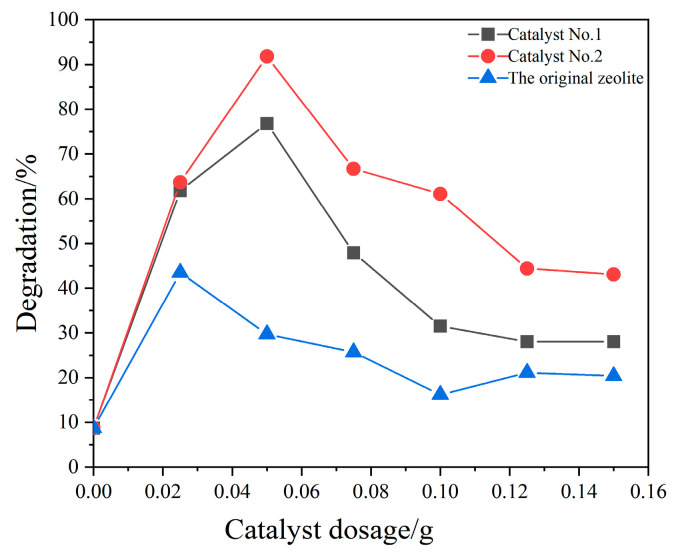
Influence of catalyst dosage (T = 20 °C, UV = 2 lamps (a lamp power of 8 w), flow rate = 0.5 L/min, initial concentration = 3.313 g/m^3^).

The catalyst dosage directly affects the photocatalytic degradation rate. CQDs/clinoptilolite composites were investigated under other optimal conditions for a single factor of catalyst dosage, as shown in Figure 10. The photocatalytic degradation of xylene was carried out at different catalyst dosages. When no catalyst was used, the degradation rate of xylene directly under the action of UV light was only 8%. When the original zeolite was used directly as the catalyst, the catalytic activity was weak and the overall degradation efficiency was slightly lower, below 25%. The xylene’s degradation efficiency showed an increasing and then decreasing trend under the action of Catalyst No. 1 and Catalyst No. 2. The catalytic efficiency was the highest when the catalyst was 0.05 g, and the degradation rates were 91% and 72%, respectively. The results showed that Catalyst No. 2 had the best degradation effect on xylene gas, and the degradation efficiency of xylene was significantly improved after the loading of CQDs on the clinoptilolite compared with the original zeolite.

**Figure 11:**
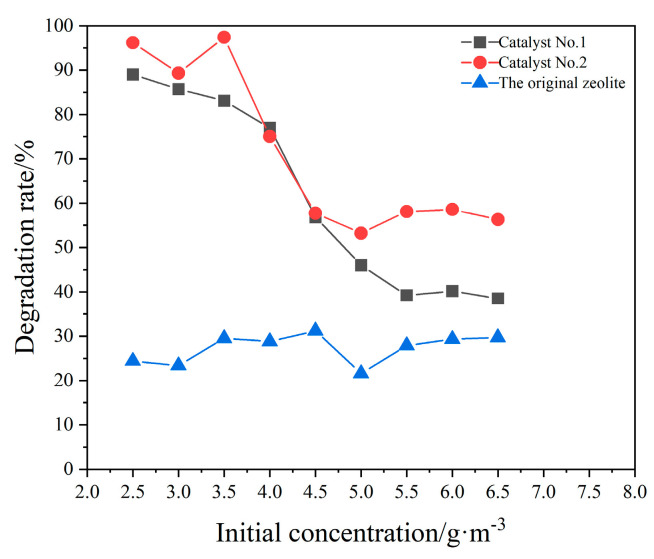
Influence of initial concentration (T = 20 °C, UV = 2 lamps (a lamp power of 8 w), catalyst dosage = 0.05 g).

Influence of initial concentration on the degradation efficiency of xylene. CQDs/clinoptilolite composites were investigated under other optimal conditions for a single factor of initial concentration, as shown in Figure 11. The photocatalytic degradation of xylene was carried out at different initial concentrations. As the input concentration of xylene increased, for catalyst No. 2, the degradation efficiency first decreased and then leveled off. Catalyst No. 1 and Catalyst No. 2 reached maximum degradation rates of 89% and 92% at an initial concentration of 2.5 g/m^3^, respectively. The degradation curves showed a decreasing trend. In contrast, the degradation effect was poorer when the original zeolite was used as the catalyst and the degradation curve showed a smooth trend.

**Figure 12:**
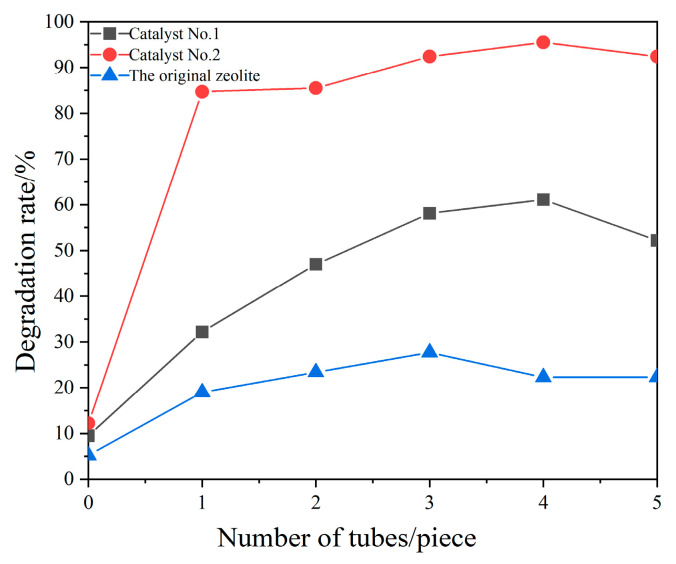
Influence of number of tubes (T = 20 °C, UV = 2 lamps (a lamp power of 8 w), catalyst dosage = 0.05 g).

Light intensity is a necessary condition for photocatalytic degradation of pollutants, and the ease of achieving the required conditions for photocatalysis in practical applications determines whether the method and materials can be widely promoted and used [37]. CQDs/clinoptilolite composites were investigated under other optimal conditions for single factors of light intensity, as shown in Figure 12. The experiments were conducted with the number of lamps from one to five, respectively. The xylene degradation rate showed a trend of increasing first and then stabilizing. When there was no light, the degradation rates of Catalyst No. 1, Catalyst No. 2, and the original zeolite were 11%, 10%, and 5%, respectively. With the increase in light intensity, the degradation rates of Catalyst No. 1 and Catalyst No. 2 in the experiment reached the maximum with four lamps, reaching 60% and 95%, respectively; the degradation rate of the original zeolite reached the maximum at three lamps, reaching 25%. The results showed that the catalytic performance of the Catalyst No. 2 composite was significantly better than that of Catalyst No. 1 and the original zeolite.

In summary, the optimum degradation conditions for the whole experiment were that the catalyst was Catalyst No. 2, the residence time was 90 s, the initial concentration was 2.5 g/m^3^, the light intensity was three lamps for irradiation, and the catalyst dosage was 0.05 g.

### 3.3. Catalyst Lifetime

**Figure 13:**
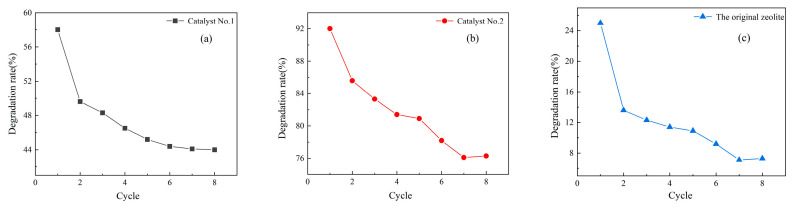
Catalyst lifetime of (**a**) Catalyst No. 1, (**b**) Catalyst No. 2, (**c**) the original zeolite.

In the system of the photocatalytic degradation of xylene, whether the catalyst still has excellent catalytic activity after several uses is particularly important for the study of organic waste gas degradation in practical applications [38]. For this reason, the recyclability of Catalyst No. 1, Catalyst No. 2, and the original zeolite was analyzed under optimal conditions. The results are shown in Figure 13a–c, and the degradation rates of all three catalysts were gradually decreased until they were stable with the increase in the number of tests under the optimal conditions. Catalyst No. 2 had better stability and should be the optimal ratio of glucose to clinoptilolite in the synthesis of materials, with a large amount of carbon quantum dots introduced, and the degradation rate was stable at about 78% after eight repetitions. The stability of Catalyst No. 1 was average, and the degradation rate was stable at about 43% after eight repetitions. The stability of the original zeolite was very poor, and the original zeolite did not have the introduction of carbon quantum dots. The original zeolite mainly plays an adsorption role for xylene gas, and only a 7% degradation rate was achieved after repeated use to reach adsorption saturation. As per the reports, CQDs have also shown long-term stability up to one month under ambient conditions indicating CQDs’ storage is not critical and they can be preserved for a long period of time before their use [39]. CQDs have excellent long-life stability, and very high thermal stability and biocompatibility [40,41]. 

### 3.4. Mechanistic Analysis of Photocatalytic Performance

In order to investigate the photocatalytic mechanism of CQDs/clinoptilolite composites, experiments on the capture of active substances in the xylene degradation reaction were conducted [42]. In the degradation of xylene by CQDs/clinoptilolite composites, the capture agent was added to the composites.

The experimental results are shown in Table 4:

The photocatalytic degradation of xylene was significantly inhibited, and ·OH and ·O_2_^−^ were the effective active substances in the degradation process. In the degradation experiment, the degradation rate of xylene was 90%, and the removal rate of xylene was 19% when ·O_2_^−^ was captured and 23% when ·OH was captured.

From the previous XPS characterization analysis, the prepared carbon quantum dots produce a large number of C=O double bonds and carboxyl functional groups attached during the aromatization process. The oxygen atom in the carbon oxygen double bond has a strong electron-absorbing effect, and the carboxyl group as an electron-absorbing group can accept the electrons generated by light. Obviously, the main function of CQDs is to inhibit the compounding of photogenerated electrons.

It can be seen from the solid diffuse reflection spectrum that the solid diffuse reflection curves of Catalyst No. 2, Catalyst No. 1, and the original zeolite are tangential by the truncation method, and the intersection point of the extension line of the tangent and the x-axis is the absorption wavelength threshold, which is substituted into the formula Eg = 1240/λ. It was found that after clinoptilolite was loaded by CQDs, the band gap gradually narrowed as the absorption wavelength increased. This improves the absorption of light by the catalyst and facilitates the separation of photogenerated electron-hole pairs [43,44].

Clinoptilolite is dominated by SiO_2_, followed by Al_2_O_3_, and contains small amounts of TiO_2_, Fe_2_O_3_, FeO, MgO, CaO, and other metal oxides. The surface of carbon quantum dots located in CQDs/clinoptilolite composites has functional groups such as hydroxyl groups and carboxyl groups, which have the effect of inhibiting photogenerated electron recombination. The clinoptilolite is loaded with CQDs, which induce the formation of electron-hole pairs of SiO_2_ in the clinoptilolite to absorb oxidants or reducing agents to generate reactive radicals and oxidize xylene gas [45]. In catalytic degradation reactions, photogenerated electrons react with O_2_ in air to generate ·O_2_^−^, while holes trap H_2_O molecule formation ·OH [46]. When xylene gas comes into contact with the composites, ·O_2_^−^ and ·OH oxidizes xylene gas to produce carbon dioxide and water.

## 4. Conclusions

In this study, carbon quantum dots were used to modify clinoptilolite to make composite materials. The composite materials were used for the degradation of xylene gas, and the adsorption and degradation performance of the materials, and the influencing factors and their mechanisms, were compared and investigated. The catalytic effects proved that the degradation rate of Catalyst No. 2 was better than that of Catalyst No. 1 and the original zeolite under any conditions, with a maximum degradation rate of 97.4% under optimal conditions, and the stability was still good after eight cycles of use. The results of radical capture experiments, combined with the characterization analysis of the composites, revealed that a large number of oxygen-containing functional groups such as hydroxyl and carboxyl groups on the surface of CQDs in the composites play an important role in the photocatalytic degradation process. This work shows that the CQDs/clinoptilolite can effectively remove the refractory organic pollutants, and it is of great significance to the research on its realization and applicability in practice. Carbon-based quantum dots are mainly composed of common non-toxic elements, and are not only environmentally friendly but also easy to prepare. Thus, the wide application of carbon-based quantum dots is in line with the current concept of the carbon cycle and the green sustainable social concept [47]. Therefore, CQDs will play a significant role in future photocatalytic devices.

## Figures and Tables

**Table 1:** Content of prepared components of different materials.

Material Name	Glucose Dosage/mol	Clinoptilolite Mass/mol
The original zeolite	0	5.00 × 10^−3^
Catalyst No. 1	6.88 × 10^−3^	5.00 × 10^−3^
Catalyst No. 2	1.38 × 10^−2^	5.00 × 10^−3^

**Table 2:** Elemental content of Catalyst No. 2.

Element	Percentage by Weight (%)	Percentage of Atoms (%)
C K	0.72	13.98
O K	4.29	62.52
Al K	0.13	1.16
Si K	2.61	21.68
K K	0.11	0.66

**Table 3:** Elemental atomic percentage of Catalyst No. 1 and Catalyst No. 2.

Element	Atomic Percentage of Catalyst No. 1 (%)	Atomic Percentage of Catalyst No. 2 (%)
Al 2p	6.36	4.91
Si 2p	18.75	14.01
C 1s	19.99	37.96
K 2p	0.38	0.22
O 1s	54.52	42.91

**Table 4:** Active substance capture experiment.

Capture Agent	None	TEMPO	P-Benzoquinone
Capture of active substances		·OH	·O_2_^−^
Xylene degradation rate/%	90	23	19

## Data Availability

All data are provided in the manuscript.
